# Stem cell therapy for stroke: mechanisms, clinical translation, and future perspectives

**DOI:** 10.3389/fncel.2026.1801759

**Published:** 2026-06-22

**Authors:** Yu Xia, Juan Jin, Lei Song, Zekai Hu, Jue Wang, Tingting Meng, Jie Zhuang

**Affiliations:** 1 Department of Rehabilitation Medicine, The Second Rehabilitation Hospital of Shanghai, Shanghai, China; 2 School of Special Education and Rehabilitation, Binzhou Medical University, Yantai, Shandong, China

**Keywords:** neural repair, regenerative medicine, stem cell therapy, stroke, treatment

## Abstract

Stroke remains a primary global driver of death and disability. Although acute revascularization therapies (intravenous thrombolysis and mechanical thrombectomy) have advanced, their narrow therapeutic windows result in persistent neurological deficits for many survivors. This necessitates restorative treatments during the subacute and chronic phases. Consequently, stem cell therapies (SCT) have become a central strategic focus, moving beyond the early hypothesis of direct cell replacement toward modulation of the post-stroke microenvironment. This review synthesizes the mechanistic evidence for various stem cells, primarily mesenchymal stem cells and neural stem cells, and evaluates the clinical landscape, which shows reliable safety but variable efficacy. Critically, the review addresses a fundamental translational barrier: the poor post-transplantation survival of stem cells, a limitation that has become a central focus of ongoing research. The discussion further addresses the paradigm shift toward cell-free therapies (e.g., extracellular vesicles) and innovative bioengineering strategies aimed at enhancing cell potency. Realizing the full clinical potential of these therapies for stroke requires addressing persistent challenges in standardization and translation through dedicated collaborative research.

## 1 Introduction

Stroke, a leading global cause of death and adult disability, arises from the acute disruption of cerebral blood flow, most commonly ischemic (approximately 85%) due to occlusion or hemorrhagic due to vessel rupture (Choudhery et al., 2025; Guo et al., 2021). While acute reperfusion therapies, such as intravenous tissue plasminogen activator (tPA) and endovascular thrombectomy, have revolutionized outcomes by targeting the salvageable ischemic penumbra, their utility remains severely restricted by narrow time windows and contraindications (Yang et al., 2025). Furthermore, these perfusion-centric approaches fail to mitigate the protracted cascade of secondary injury (e.g., excitotoxicity, oxidative stress, blood-brain barrier disruption) that expands tissue damage or address inherent limitations in adult CNS regeneration (Li et al., 2021; Sarmah et al., 2024). This therapeutic gap is particularly pronounced in hemorrhagic stroke, where specific neurorestorative options are virtually non-existent (Kaur et al., 2023). Consequently, the imperative is now shifting toward developing treatments that promote active repair, plasticity, and functional recovery in the subacute and chronic phases. Stem cell-based therapies (SCT) have thus emerged as a preeminent candidate. Although initially hypothesized to replace lost cells via direct differentiation, particularly for neural stem cells, evidence now overwhelmingly supports paracrine effects as the primary therapeutic mechanism for mesenchymal stem/stromal cells (MSCs) (Rahimi Darehbagh et al., 2024; Weber et al., 2025). Acting as multifaceted “bioreactors,” SCT secrete a complex array of bioactive molecules–including neurotrophic factors, cytokines, and nucleic acids encapsulated within extracellular vesicles (EVs)–that orchestrate a conducive microenvironment. This potent secretome both mitigates secondary injury and enhances endogenous repair, fostering structural and functional plasticity (Berlet et al., 2022; Zhang et al., 2022).

This comprehensive narrative review therefore aims to provide a critical synthesis of the current state of SCT for stroke. We will systematically dissect the complex multi-mechanistic framework underlying stem cell benefits, moving beyond mere cataloging to discuss integrated signaling pathways. Furthermore, we critically appraise the heterogeneous clinical trial landscape, explore the transformative potential of EV-based, cell-free therapeutics, and delve into cutting-edge bioengineering approaches (e.g., genetic modification, preconditioning) designed to overcome existing limitations. By integrating these insights, we seek to chart the arduous path from preclinical promise to optimized, standardized clinical reality for stroke patients globally.

## 2 Mechanisms of action: an integrated multi-mechanistic framework of repair beyond replacement

**FIGURE 1:**
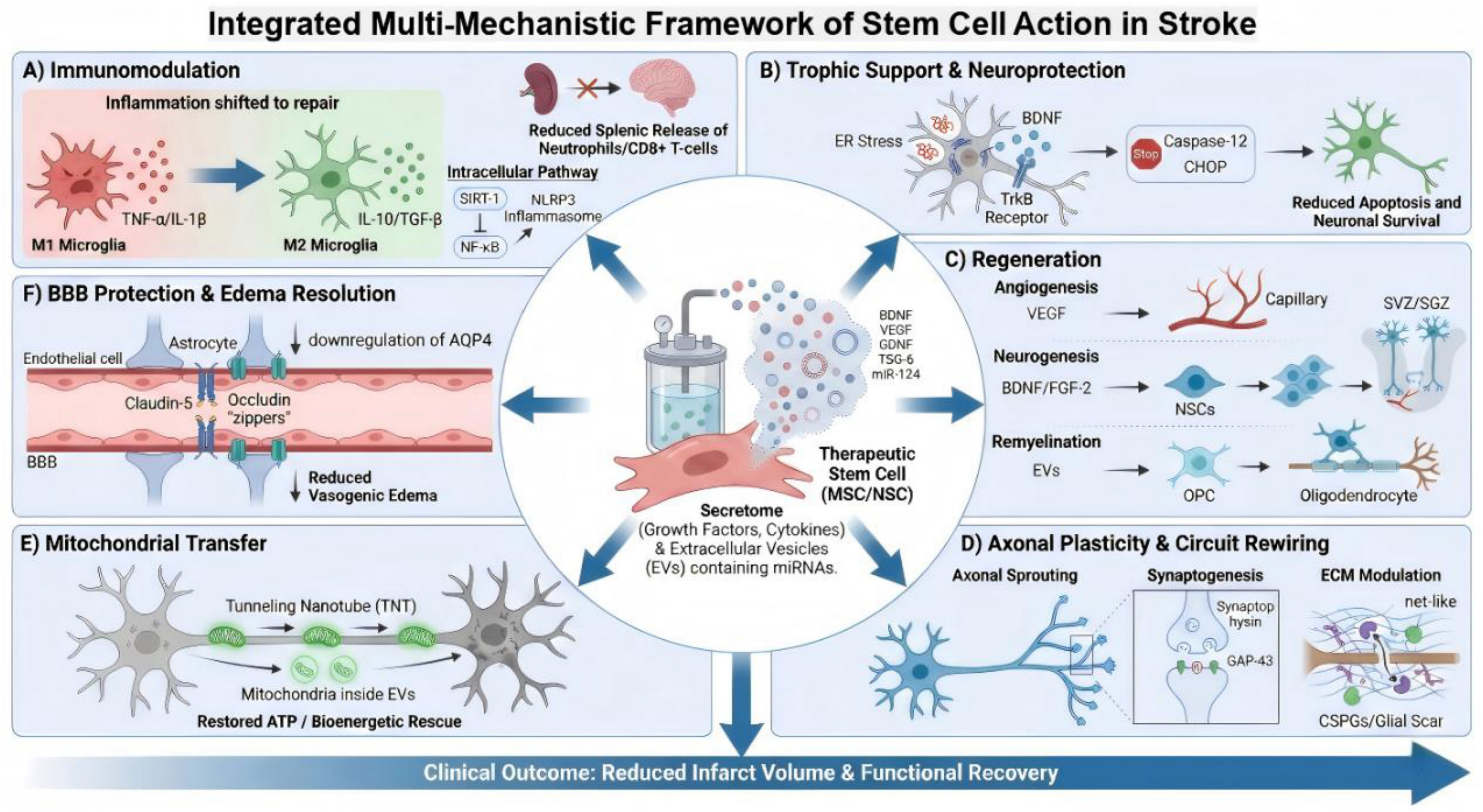
Integrated multi-mechanistic framework of stem cell action in stroke. Therapeutic MSCs function as biological factories, releasing a secretome of growth factors (e.g., BDNF, VEGF) and extracellular vesicles. This “coordinated signaling network” orchestrates tissue repair through six synergistic mechanisms: **(A)** immunomodulation shifting microglia from inflammatory (M1) to reparative (M2) states; **(B)** trophic support inhibiting neuronal apoptosis; **(C)** stimulation of angiogenesis, neurogenesis, and remyelination; **(D)** enhancement of axonal plasticity and synaptic rewiring; **(E)** bioenergetic rescue via mitochondrial transfer; and **(F)** stabilization of the blood-brain barrier to reduce edema. Collectively, these pathways reduce infarct size and promote functional recovery.

The therapeutic action of stem cells in stroke constitutes a coordinated, multi-mechanistic response targeting the complex, multifactorial pathophysiology of injury (see Figure 1).

However, a critical appraisal of the literature reveals that these mechanisms are not equally weighted, operate on distinct temporal axes, and function in a highly interdependent, rather than redundant, manner. While the six pathways outlined below are often presented in parallel, emerging evidence suggests a hierarchical and sequential organization. Immunomodulation and blood-brain barrier (BBB) protection appear to act as dominant early-phase “gatekeeper” mechanisms, establishing a permissive microenvironment in the absence of which subsequent neurotrophic support, angiogenesis, and plasticity are substantially blunted. Mechanistically, the failure of many acute-phase clinical trials (e.g., TREASURE) may stem from attempting to deliver trophic or pro-plasticity signals into a hostile inflammatory milieu before this gatekeeper function has been established. Conversely, in the subacute and chronic phases, the relative contribution shifts toward trophic support, angiogenesis/neurogenesis, and axonal plasticity, with mitochondrial transfer representing a distinct, direct bioenergetic rescue mechanism that may operate in parallel. Critically, these mechanisms are not redundant but synergistic: immunomodulation reduces oxidative stress, thereby preserving the efficacy of secreted neurotrophic factors; neovascularization provides both perfusion and a physical scaffold for migrating neural progenitors; and enhanced axonal plasticity requires both trophic support and the removal of inhibitory matrix components. The following sections critically evaluate the evidence base for each mechanism, with emphasis on the strength of causal linkage to functional recovery and the temporal context in which each mechanism predominates.

### 2.1 Immunomodulation and the reshaping of neuroinflammation

Post-stroke neuroinflammation is biphasic: an immediate, controlled response clears debris, but a sustained, dysregulated reaction–driven by activated resident microglia and infiltrating peripheral immune cells (neutrophils, monocytes/macrophages, lymphocytes)–is a potent promoter of secondary neurodegeneration and repair inhibition (Anthony et al., 2022). Among all stem cell mechanisms, particularly for MSCs, immunomodulation carries the strongest weight of evidence as a primary, non-redundant early mediator of efficacy. Its critical importance is underscored by three observations: first, mesenchymal stem/stromal cells (MSCs) exert their most potent effects on functional recovery when administered during the subacute inflammatory peak rather than the hyperacute phase; second, selective ablation of the immunomodulatory capacity (e.g., via genetic disruption of TNF-stimulated gene 6 protein (TSG-6) or prostaglandin E2 (PGE2) synthesis) abrogates therapeutic benefit even when trophic factor secretion remains intact; and third, the therapeutic window for immunomodulation closes as the inflammatory response transitions from active modulation to fibrotic scar consolidation.

Mesenchymal stem/stromal cells (MSCs) are potent immunomodulators that actively shift inflammation toward a reparative phenotype, primarily through microglia and macrophage polarization. MSCs and their secretome reprogram these cells from the cytotoxic M1 phenotype (releasing TNF-α, IL-1β, and ROS) to the phagocytic, pro-repair M2 state (secreting IL-10, TGF-β, and growth factors) (Berlet et al., 2022). Key mediators include MSC-derived PGE2, TSG-6, and extracellular vesicles (EVs) carrying specific microRNAs (e.g., miR-124, miR-146a) (Chang C. et al., 2025; Nakano and Fujimiya, 2021). However, the translational relevance of this polarization axis remains debated, as human microglia exhibit greater phenotypic heterogeneity and less binary polarization than murine models suggest.

Recognizing that stroke triggers profound systemic immunodepression and splenic activation, intravenously administered MSCs modulate this crucial peripheral response by homing to lymphoid organs. This action limits the infiltration of damaging neutrophils and cytotoxic CD8+ T cells while concurrently promoting anti-inflammatory regulatory T cell (Treg) expansion (McMillan et al., 2026; Sarmah et al., 2022). This systemic immunomodulatory arm may be particularly important for intravenous delivery, where direct brain engraftment is minimal, and constitutes a distinct mechanistic pathway separable from local intracerebral effects. Furthermore, MSCs inhibit key inflammatory pathways. For instance, intra-arterial delivery upregulates the deacetylase Sirtuin-1 (SIRT-1), which subsequently inhibits the nuclear factor kappa B (NF-κB) pathway. This cascade downregulates the NLR family pyrin domain containing 3 (NLRP3) inflammasome–a central mediator of IL-1β maturation and pyroptosis–and associated components, thereby mitigating caspase-1 activation and cellular apoptosis (Sarmah et al., 2022, 2024). Despite this compelling preclinical cascade, direct evidence linking inflammasome suppression to functional outcome improvements in large-animal or human studies remains limited, representing a critical gap.

### 2.2 Trophic support and paracrine-mediated neuroprotection

The stem cell secretome serves as a rich source of neurotrophic and growth factors essential for neuronal survival, axonal growth, and synaptic plasticity, and this paracrine support is arguably the dominant mechanism for mesenchymal stem/stromal cells (MSCs). However, the relative contribution of trophic support compared to immunomodulation remains a subject of active investigation. Evidence from conditioned media and EV studies demonstrates that trophic factors can exert neuroprotection even in the absence of live cells, suggesting that this mechanism is sufficient, though not necessarily necessary, for some degree of efficacy. The weight of evidence currently supports a model where trophic support acts as a secondary wave mechanism: after immunomodulation has attenuated the cytotoxic microenvironment, secreted neurotrophins can effectively engage survival pathways that would otherwise be overwhelmed by oxidative stress and inflammatory signaling. Central to post-stroke recovery is the brain-derived neurotrophic factor (BDNF) and its high-affinity receptor, Tropomyosin receptor kinase B (TrkB). BDNF promotes neuronal survival, dendritic arborization, and synaptic strength. Research indicates that intra-arterial MSCs significantly upregulate BDNF and TrkB expression in the peri-infarct cortex. This activated BDNF/TrkB signaling cascade is crucial for mitigating endoplasmic reticulum (ER) stress, a major contributor to delayed neuronal apoptosis. By reducing the expression of ER stress markers [glucose-regulated protein 78 (GRP78) and CAAT/enhancer-binding protein (C/EBP) homologous protein (CHOP)] and downstream apoptotic executors like caspase-12, BDNF signaling helps preserve vulnerable neurons in the ischemic penumbra (Kaur et al., 2021, 2023). Notably, the BDNF pathway exemplifies mechanistic synergy: its anti-apoptotic efficacy is substantially enhanced when combined with prior immunomodulation that reduces oxidative stress, which would otherwise impair TrkB signaling. This interdependence challenges the notion of trophic support as an independent mechanism. Beyond BDNF, stem cells secrete a multifaceted cocktail of factors that provide comprehensive support to the injured neurovascular unit. This includes vascular endothelial growth factor (VEGF) for angiogenesis, glial cell line-derived neurotrophic factor (GDNF) for dopaminergic and motor neuron survival, nerve growth factor (NGF), and basic fibroblast growth factor (bFGF) (Hao et al., 2025).

### 2.3 Promotion of angiogenesis, neurogenesis, and oligodendrogenesis

Post-stroke recovery requires vascular niche restoration, neurogenesis, and axonal remyelination–processes substantially mediated by stem cells. The causal contribution of these processes to functional recovery, however, requires careful scrutiny. While angiogenesis and neurogenesis robustly correlate with improved outcomes in preclinical models, direct evidence that stem cell-induced neurogenesis *per se*, rather than concurrent trophic or immunomodulatory effects–drives recovery remains circumstantial. Studies using selective inhibitors of neurogenesis (e.g., temozolomide) suggest that while endogenous neurogenesis contributes to recovery, it accounts for only a fraction of the total treatment effect. Functionally, the vascular effects may be more critical: neovascularization not only restores perfusion but also provides a structural scaffold for migrating neural progenitors and secretes niche factors that support neuronal survival. The relative contribution of each process likely depends on lesion topography, with white matter stroke models showing greater dependence on oligodendrogenesis and remyelination. Stem cells promote angiogenesis via the secretion of factors like vascular endothelial growth factor (VEGF), which drives endothelial proliferation and migration, thereby inducing neovascularization within the ischemic border zone. This neovascularization enhances perfusion and provides a physical scaffold along with signaling cues for migrating neural progenitor cells (NPCs) (Habib and Steinberg, 2025). Neurogenesis takes place within the adult brain’s intrinsic niches: the subventricular zone (SVZ) and the subgranular zone (SGZ) of the hippocampal dentate gyrus. Stem cell-derived factors (BDNF, VEGF, and FGF-2) stimulate endogenous neural stem/progenitor cell (NSC/NPC) proliferation within these niches, facilitating subsequent migration toward the injury site. Although the survival and functional integration of these endogenously generated neurons remain debatable, they nonetheless contribute to the recovering brain’s pool of plastic elements (Tang et al., 2023). Crucially, stem cells also support oligodendrogenesis and remyelination, processes vital for repairing white matter injury and reversing axonal demyelination. Specifically, MSC-conditioned media and extracellular vesicles (EVs) promote the differentiation of oligodendrocyte precursor cells (OPCs) into mature, myelinating oligodendrocytes, thereby facilitating axonal remyelination and improving neural conduction velocity (Park and Borlongan, 2021). White matter repair has emerged as a particularly underappreciated mechanism, with recent data suggesting that functional recovery in large animal models correlates more strongly with remyelination than with neurogenesis.

### 2.4 Enhancement of axonal plasticity and circuit reorganization

Functional recovery following stroke is driven by the brain’s capacity for neural reorganization, a process stem cells foster by promoting synaptic and axonal plasticity. This effect is demonstrated by the therapy’s ability to upregulate key markers [e.g., GAP-43, synaptophysin, spinophilin] in the peri-infarct cortex, thereby confirming robust axonal sprouting and synaptogenesis essential for neural circuit remapping (Kaur et al., 2023). This mechanism carries particular importance in chronic stroke, where neuroprotection is no longer relevant, and the therapeutic goal shifts entirely to circuit remodeling. The evidence for axonal plasticity as a primary mechanism in the chronic phase is stronger than in acute settings, supported by studies demonstrating that stem cell administration months after injury can still drive synaptic reorganization and functional gains. However, the specificity of this plasticity remains a concern: uncontrolled axonal sprouting can theoretically lead to maladaptive plasticity, including aberrant circuit formation or hyperexcitability. The field currently lacks long-term safety data on the functional quality of stem cell-induced rewiring. Beyond this direct neuroplasticity, stem cells also counteract the inhibitory post-injury extracellular matrix. The resulting glial scar–formed primarily of reactive astrocytes and chondroitin sulfate proteoglycans (CSPGs)–constitutes a critical impediment to axonal growth. The stem cell secretome modulates astrocyte reactivity and increases CSPG-degrading enzyme expression, thereby creating a permissive milieu for axonal regeneration. The relative contribution of active growth promotion versus inhibitory matrix degradation to net plasticity remains an open question, with some evidence suggesting that matrix modulation may be the rate-limiting step for sustained axonal extension.

### 2.5 Mitochondrial transfer and bioenergetic rescue

Stem cell-mediated recovery relies on direct cell-to-cell communication to achieve bioenergetic rescue, counteracting the profound mitochondrial dysfunction in post-stroke neurons that initiates ATP depletion, calcium overload, and apoptotic signaling. Mitochondrial transfer represents a conceptually distinct mechanism from paracrine signaling, as it involves direct organelle donation rather than soluble factor secretion. Its relative importance remains controversial. While elegant studies using fluorescent labeling and tunneling nanotube (TNT) visualization demonstrate that this phenomenon occurs *in vitro* and *in vivo*, quantitative assessments suggest that only a minority of peri-infarct neurons receive transferred mitochondria. The functional impact may therefore be highly localized rather than widespread. Furthermore, the extent to which mitochondrial transfer contributes independently of concurrent paracrine effects remains unresolved, as most studies cannot isolate these pathways. Nevertheless, this mechanism is mechanistically unique in providing immediate bioenergetic support rather than triggering host repair pathways, making it particularly relevant for acute neuroprotection. The first key mechanism is direct mitochondrial transfer: mesenchymal stem/stromal cells (MSCs) form tunneling nanotubes (TNTs) with damaged neurons, enabling the direct transfer of healthy organelles. This donation effectively rescues neuronal bioenergetics, restores membrane potential, and suppresses apoptosis (Hamblin and Lee, 2021; Sarmah et al., 2024). A second route, designated “horizontal mitochondrial transfer,” delivers mitochondria or their components within extracellular vesicles (EVs)–a method cited as a more efficient and safer delivery mode. This process is governed by a sophisticated molecular axis: Sirtuin-1 (SIRT-1) promotes mitochondrial biogenesis, while mitochondrial Rho GTPase 1 (RHOT-1/Miro1) is essential for anchoring mitochondria to microtubules for transport (Sarmah et al., 2024). Furthermore, beyond physical donation, stem cell-derived factors can induce metabolic reprogramming in injured neurons. For instance, induced pluripotent stem cell-derived MSCs (iMSCs) boost neuronal mitochondrial respiration and glycolysis, thus enhancing overall metabolic activity *in vitro* and *in vivo* (Kawatani et al., 2025). Distinguishing between direct mitochondrial transfer and metabolic reprogramming induced by secreted factors remains a methodological challenge that limits mechanistic interpretation.

### 2.6 Blood-brain barrier protection and edema resolution

The disruption of the BBB following stroke leads to vasogenic edema, hemorrhagic transformation, and the influx of toxic plasma components, processes that stem cell therapy can help mitigate. BBB protection ranks alongside immunomodulation as a critical early “gatekeeper” mechanism. Evidence from both preclinical and clinical studies indicates that BBB integrity at baseline predicts response to stem cell therapy, and treatment efficacy is substantially reduced when administered after BBB disruption has progressed to irreversible structural damage. Mechanistically, BBB protection may be both a direct effect, via tight junction stabilization and modulation of aquaporin-4 (AQP4); and an indirect consequence of reduced neuroinflammation, as inflammatory cytokines are major drivers of barrier breakdown. This interdependence again illustrates the difficulty of isolating individual mechanisms. A key target is the regulation of Aquaporin-4 (AQP4), a water channel whose upregulation on astrocytic endfeet exacerbates post-stroke edema. Intra-arterial mesenchymal stem/stromal cell (MSC) therapy has been shown to downregulate AQP4 expression. This is achieved through modulation of the protein kinase C delta/matrix metalloproteinase-9 (PKCδ/MMP-9) pathway and the Sirtuin-1/nuclear factor kappa B (SIRT-1/NF-κB) axis, thereby reducing edema and helping to preserve BBB integrity (Datta et al., 2022, 2025). Concurrently, stem cell-derived factors contribute to BBB stabilization by upregulating the expression of critical tight junction proteins–such as claudin-5, occludin, and zonula occludens-1 (ZO-1)–which helps reseal the compromised barrier. Despite robust preclinical data, clinical translation of BBB protection as a primary endpoint remains limited, as current imaging modalities lack the resolution to quantify regional BBB integrity with sufficient sensitivity for use as a surrogate marker in cell therapy trials.

### 2.7 The challenge of transplanted cell survival: a critical bottleneck

Despite the multifaceted therapeutic mechanisms described above, a fundamental limitation pervades the field: most transplanted cells undergo rapid apoptosis or clearance within days to a few weeks after administration. This poor survival is driven by the same hostile post stroke microenvironment that stem cells aim to modulate, including oxidative stress, inflammatory cytokines, and matrix disruption. For mesenchymal stem/stromal cells delivered intravenously, the pulmonary first pass effect further compounds this loss. Consequently, therapeutic benefits arise from a transient paracrine action rather than sustained cellular residence. This temporal disconnect underscores the need for strategies that enhance cell persistence, such as preconditioning, genetic engineering, or scaffold based delivery.

## 3 Stem cell sources and delivery considerations: defining the therapeutic protocol

The therapeutic efficacy of a stem cell product hinges upon its cellular source and delivery methodology. Optimization of these critical variables constitutes a principal focus of translational research.

### 3.1 Cell types: a comparative analysis

In the clinical translation of stem cell therapy for stroke, the selection of cell type is one of the core factors determining therapeutic potential. Currently, the most widely used cell type is mesenchymal stem/stromal cells (MSCs), which possess multipotent differentiation capacity *in vitro*, although their therapeutic effects in stroke are primarily attributed to paracrine and immunomodulatory actions rather than direct neuronal replacement. Although capable of differentiating into mesodermal lineages, their primary therapeutic effects in stroke are attributed to their rich secretome and immunomodulatory functions. MSCs exhibit low immunogenicity, expressing minimal levels of Major Histocompatibility Complex (MHC) class II molecules and co-stimulatory markers, making them suitable for allogeneic transplantation without the need for rigorous immunosuppression and facilitating “off-the-shelf” treatment. They can be isolated from various tissues, including bone marrow (BM-MSCs), adipose tissue (AD-MSCs), Wharton’s jelly or blood from the umbilical cord (UC-MSCs), and dental pulp (DPSCs). Among these, UC-MSCs and AD-MSCs are often preferred due to their relative ease of harvest and higher proliferative capacity (Choudhery et al., 2025; Shen et al., 2024), while DPSCs have also demonstrated particular neuroprotective potential in preclinical models of hemorrhagic stroke (He et al., 2025). Extensive clinical data support their favorable safety profile.

Key cellular candidates include neural stem/progenitor cells (NSCs/NPCs) and bone marrow mononuclear cells (BMMNCs). Committed to the neural lineage, NSCs/NPCs offer robust potential for direct neuronal replacement and synaptic integration into host circuits. Their derivation routes include fetal central nervous system (CNS) tissue (a route with ethical and logistical challenges), differentiation from induced pluripotent stem cells (iPSCs), and the direct reprogramming of somatic cells into induced NSCs (iNSCs) or neurons (Wang S. et al., 2024). Despite this potential for structural repair, NSC/NPCs carry a higher theoretical tumorigenic risk, a concern that demands rigorous scrutiny in clinical translation. This risk is primarily attributed to the presence of residual undifferentiated proliferative cells within the transplanted population that, under the influence of the post-stroke inflammatory and trophic microenvironment, could theoretically undergo uncontrolled proliferation and form teratomas or other neoplasms. The risk is particularly elevated when using pluripotent stem cell-derived products (e.g., from iPSCs) where incomplete differentiation or genetic instability may persist. Therefore, unlike mesenchymal stem/stromal cells (MSCs), which have a well-documented long-term safety profile, the clinical application of NSCs/NPCs necessitates stringent quality control measures, including the implementation of advanced cell sorting protocols to eliminate residual undifferentiated cells, the use of suicide gene strategies as a safety switch, and extended post-transplantation surveillance in clinical trial participants and often necessitate precise, localized delivery. Conversely, BMMNCs are a heterogeneous, unfractionated population comprising hematopoietic stem cells (HSCs), mesenchymal stromal cells (MSCs), endothelial progenitor cells (EPCs), and diverse immune cells. Autologous BMMNC transplantation minimizes immune rejection and simplifies regulatory pathways. Several early-phase stroke trials have employed autologous BMMNCs, with network meta-analyses (NMAs) suggesting efficacy in improving motor function and activities of daily living (Chang W. et al., 2025; Habib and Steinberg, 2025).

Induced pluripotent stem cell-derived MSCs (iMSCs) constitute a critical novel source. Generated via iPSC differentiation into an MSC-like population, iMSCs integrate the unlimited expandability of iPSCs with MSC therapeutic attributes and a favorable safety profile. Crucially, this methodology demonstrates efficacy equivalent to primary MSCs in animal stroke models (Arakawa et al., 2023), thereby potentially facilitating the establishment of highly standardized, clinical-grade cell banks (Kawatani et al., 2025).

### 3.2 Delivery routes: navigating the path to the brain

Delivery route profoundly impacts cell distribution, engraftment, safety, and mechanism of action. Intravenous (IV) administration is the least invasive route, offering easy administration and the capacity to modulate systemic inflammation, particularly via splenic interaction. The major drawback, however, is the “pulmonary first-pass effect,” which traps the majority of cells, especially larger mesenchymal stem/stromal cells (MSCs), in lung capillaries; consequently, only a tiny fraction (<1%–2%) reaches the cerebral vasculature. This low yield necessitates higher doses and risks systemic side effects (Achón Buil et al., 2023). In contrast, intra-arterial (IA) injection, which delivers cells directly into the internal carotid or middle cerebral artery, achieves more targeted delivery to the affected hemisphere and higher initial cerebral engraftment compared to the IV route. It requires stringent control of cell size and infusion parameters to mitigate micro-emboli risk. Preclinical efficacy is often highlighted by the higher localized cell concentration near the injury, suggesting it is better suited for targeting the acute/subacute phase (Kaur et al., 2021; Sarmah et al., 2022).

Intracerebral (IC) or intraparenchymal delivery, the most invasive technique, utilizes stereotactic injection directly into the infarct cavity or surrounding parenchyma. This maximizes local cell density, proving ideal for integrating cells such as neural stem/progenitor cells (NSCs/NPCs). However, this inherent invasiveness risks secondary focal injury, hemorrhage, and inflammation; thus, IC delivery is typically reserved for chronic stroke patients with established cavities (Kawabori et al., 2024). Conversely, intrathecal (IT) injection into the lumbar subarachnoid space introduces cells directly into the cerebrospinal fluid (CSF), facilitating circulation and potential migration to cerebral perivascular spaces. Although potentially offering better brain distribution than intravenous (IV) delivery, a comparative clinical trial using umbilical cord MSCs, while finding both effective, demonstrated that IV administration exhibited a superior adverse event profile (Nguyen et al., 2025). Finally, intranasal (IN) delivery is a non-invasive technique using cell suspensions or secreted products delivered via drops or sprays. It is hypothesized that IN administration utilizes olfactory and trigeminal nerve pathways, thereby bypassing the blood-brain barrier (BBB). Despite this appeal, low efficiency in delivering therapeutic agents to deeper brain structures suggests IN is best optimized for administering cell-derived secretomes or extracellular vesicles (EVs) (Berlet et al., 2022; Jiang et al., 2025).

### 3.3 Timing of administration: a critical therapeutic variable

The optimal therapeutic window is dictated by the mechanism and the shifting post-stroke pathophysiological landscapes. Acute phase intervention (generally <24–48 h) targets penumbral rescue, excitotoxicity mitigation, and suppression of the initial inflammatory surge. However, this window is characterized by a hostile microenvironment that critically impedes transplanted cell survival. Mechanistically, the hyperacute phase exhibits peak levels of M1-polarized microglia/macrophages releasing high concentrations of TNF-α, IL-1β, and reactive oxygen species (ROS), which directly damage cell membranes and mitochondrial integrity (Anthony et al., 2022). Concurrently, pro-apoptotic pathways (e.g., caspase-3, Bax) dominate, while matrix metalloproteinases (MMP-9) disrupt the extracellular matrix, impairing cell adhesion and homing (Datta et al., 2022). In contrast, the subacute phase (days to weeks) is marked by a progressive transition toward an M2-dominant reparative phenotype, reduced oxidative stress, upregulation of pro-survival signaling (e.g., Akt, Bcl-2), and sustained expression of homing factors such as SDF-1α (Kaur et al., 2021; Sarmah et al., 2022). This evolving milieu offers a more permissive environment in which transplanted cells can exert immunomodulatory and trophic effects. The failure of the TREASURE trial, which administered allogeneic multipotent adult progenitor cells within 18–36 h post-stroke (Houkin et al., 2024), underscores the challenge of intervening during the hyperacute window. Collectively, these temporal differences in the post-stroke microenvironment likely explain why positive efficacy signals in meta-analyses predominantly derive from trials treating patients during the subacute phase (Habib and Steinberg, 2025; Osanai et al., 2025). The subacute phase (days to weeks) is the most common clinical trial window. Here, while the initial crisis stabilizes, active inflammation, apoptosis, and early plasticity persist. This environment is thus more conducive to cell survival, enabling interventions focused on modulating the subacute inflammatory response, protecting threatened tissue, and stimulating endogenous repair mechanisms. Consequently, positive signals from meta-analyses often derive from trials treating patients during this window (Habib and Steinberg, 2025; Osanai et al., 2025). In the chronic phase (months to years), the therapeutic goal shifts from neuroprotection to promoting plasticity within a stabilized brain. With the core infarction typically cystic/gliotic and inflammation low-level, therapies aim to foster axonal sprouting, remyelination, and neurogenesis to remodel existing circuits. This stability concurrently justifies more invasive delivery methods, such as intracerebral injection.

## 4 Clinical translation: navigating the bridge from bench to bedside

The clinical translation of stem cell therapy presents a fundamental complexity: despite robust preclinical success and favorable initial safety profiles, long-term efficacy remains ambiguous.

### 4.1 Safety: a consistent and reassuring finding

Dozens of Phase I/II trials (*N* = hundreds of patients) establish a robust consensus regarding the short- to medium-term feasibility and safety of autologous/allogeneic mesenchymal stem/stromal cell (MSC) and bone marrow mononuclear cell (BMMNC) transplantation. Systematic reviews and meta-analyses uniformly report no significant increase in serious adverse events (SAEs), mortality, tumorigenesis, or ectopic tissue formation compared to control groups (Xiong et al., 2024). Mild, transient adverse events (AEs)–e.g., fever or nausea–are typically infusion-related rather than cell-specific. This favorable profile is further reinforced by long-term safety monitoring, notably the 2-years results of the AMASCIS trial (involving adipose-derived MSCs) (de Celis-Ruiz et al., 2022).

### 4.2 Efficacy: a landscape of heterogeneity and modest signals

Efficacy outcomes remain highly variable, reflecting profound challenges in clinical trial design and protocol consistency in restorative neurology. The Phase 2/3 TREASURE trial exemplifies this challenge: a single intravenous dose of allogeneic multipotent adult progenitor cells (MAPCs)–a cell product distinct from MSCs with different expansion and immunomodulatory characteristics– administered to 206 patients during the hyperacute window (18–36 h), failed to meet its primary endpoint of excellent 90-days functional outcome despite a favorable safety profile. This outcome highlights how successful animal models–which often use varied cell types, timing, and routes–do not readily translate to human efficacy, particularly in this early phase (Houkin et al., 2024). Conversely, pooled meta-analyses aggregating data from numerous smaller, heterogeneous trials frequently detect a statistically significant, albeit clinically modest, benefit associated with stem cell therapy, predominantly mesenchymal stem/stromal cell (MSC)-based interventions. These analyses consistently demonstrate a shift toward favorable functional recovery: more cell-treated patients achieve improved Modified Rankin Scale (mRS, a measure of global disability) scores (e.g., mRS 0–2) at 90 days and 1 year (Osanai et al., 2025; Shen et al., 2024). Furthermore, treated groups show a greater reduction in neurological deficit, as reflected by lower National Institutes of Health Stroke Scale (NIHSS) scores (Xiong et al., 2024), alongside improvements in the Barthel Index (BI) for activities of daily living and the Fugl-Meyer Assessment (FMA) for motor function (Chang W. et al., 2025). Network meta-analyses (NMAs) offer hypothesis-generating insights by indirectly ranking the efficacy of different cell types. One NMA indicated umbilical cord MSCs (UC-MSCs) excelled for neurological recovery (NIHSS), while bone marrow mononuclear cells (BMMNCs) ranked highest for improving motor function (FMA) and daily living (BI) (Chang W. et al., 2025). A separate NMA focused on bone marrow-derived cells, ranking MSCs superior for reducing mortality and improving mRS (Wang X. et al., 2024). However, these rankings are acutely sensitive to the specific trials incorporated.

### 4.3 Beyond timing: Why preclinical success does not easily translate?

While the hostile microenvironment of the hyperacute phase is a frequently cited reason for translational failure, several additional barriers collectively limit efficacy in human stroke. Dose scaling from animal to human remains poorly characterized; preclinical studies typically use body-weight–normalized doses, but the pulmonary first-pass effect, larger blood volume, and substantially greater infarct volume in humans create a non-linear dose–response relationship, yet most clinical trials employ a single fixed dose without preceding dose-escalation studies. Patient comorbidities further complicate translation: preclinical models use young, healthy animals, whereas clinical stroke populations present with hypertension, diabetes, chronic inflammation, and atherosclerosis, all of which impair endogenous repair and compromise transplanted cell survival. Age-related regenerative decline represents another critical variable, as aging reduces neurogenic and angiogenic potential and shifts immunity toward a pro-inflammatory state, yet trials rarely stratify by age. Immune heterogeneity–beyond the autologous/allogeneic distinction–also influences response, as baseline inflammatory status and genetic variations determine how stem cells modulate the post-stroke environment. Finally, concomitant rehabilitation therapy is a major confounder: preclinical studies often incorporate structured rehabilitation that synergizes with cell therapy, but clinical trials exhibit wide variability in rehabilitation timing, intensity, and content, making it difficult to isolate the true treatment effect. Collectively, these factors, namely dose non-linearity, comorbidities, aging, immune heterogeneity, and rehabilitation variability, explain a substantial portion of the gap between preclinical promise and clinical efficacy, extending far beyond the simple narrative of “timing matters.”

### 4.4 Key therapeutic variables: a comparative analysis for clinical decision-making

The heterogeneity in clinical outcomes also stems from variability in three core therapeutic decisions: cell source, timing, delivery route, and patient selection. Regarding cell source, autologous cells eliminate immune rejection and simplify regulatory pathways, but their quality is tied to donor pathophysiology–age, chronic inflammation, and metabolic syndrome compromise potency, introducing batch-to-batch variability. Allogeneic products, particularly from umbilical cord or bone marrow of healthy donors, offer consistent, off-the-shelf availability critical for time-sensitive interventions, though even low-immunogenic MSCs may elicit responses upon repeated administration. From a clinical feasibility standpoint, allogeneic products are better suited for acute/subacute stroke, whereas autologous approaches retain value in chronic-stage personalized therapy.

Regarding timing, acute-phase administration (typically <48–72 h) aims to salvage the penumbra but faces a hostile microenvironment of reactive oxygen species and pro-inflammatory cytokines that severely compromises cell survival, as exemplified by the TREASURE trial. Chronic-phase administration (months to years) shifts the goal to promoting plasticity within a stabilized brain; the environment is more permissive for cell survival, and more invasive delivery methods become feasible. Thus, acute administration likely requires enhanced cell products or cell-free alternatives, while chronic administration benefits from patient stability and extended manufacturing time.

Delivery route selection involves trade-offs between targeting efficiency, safety, and operational complexity. Intravenous (IV) administration is minimally invasive, widely accessible, and best for systemic immunomodulation, but its low cerebral engraftment requires higher doses. Intra-arterial (IA) delivery achieves higher initial brain engraftment but requires interventional expertise and carries microemboli risk, making it suitable for subacute-phase patients in specialized centers. Intracerebral (IC) injection maximizes local cell density for integration-dependent therapies (e.g., neural stem cells) but is invasive and reserved for chronic-stage patients with established infarct cavities. Intrathecal (IT) delivery offers a middle ground with broader distribution than IV via cerebrospinal fluid, yet requires lumbar puncture expertise. Intranasal (IN) delivery is non-invasive and repeatable but shows low efficiency for delivering whole cells, making it most appropriate for extracellular vesicle–based therapies in chronic maintenance settings.

### 4.5 Sources of heterogeneity and lessons for future trials

The significant variation in trial outcomes stems from profound heterogeneity across five critical domains: product preparation (autologous vs. allogeneic source, tissue origin, passage number, and absence of standardized potency assays); dose administration (wide ranges without escalation rationale); delivery protocol (route and timing, from hours to years post-stroke); patient characteristics (age, severity, comorbidities, lesion location); and assessment methods (choice and timing of outcome measures). Furthermore, variable concomitant rehabilitation across groups confounds results. Consequently, future Phase III success requires rigorous Phase II precursors designed to identify the most promising product and protocol, alongside biomarker-driven patient selection based on immune profile or neuroimaging characteristics–a necessity underlined by *post hoc* analyses identifying younger age and earlier treatment as key predictors of MSC response (Chang et al., 2021). The preceding sections detail the substantial heterogeneity in cell sources, delivery routes, and timing windows that underpin the variable clinical outcomes observed in stem cell therapy for stroke. To facilitate direct comparison of these critical parameters across the major cell types, Table 1 integrates the key information from the previously separated tables, presenting a unified overview of cell type-specific mechanisms, delivery characteristics, optimal timing windows, major advantages, and translational challenges.

**TABLE 1:** Integrated comparative overview of stem cell therapies for stroke: cell types, delivery routes, timing, and key considerations.

Cell type	Primary source(s)	Key therapeutic mechanisms	Delivery routes and characteristics	Optimal timing window	Major advantages	Key challenges/limitations	Supporting references
Mesenchymal stem/stromal cells (MSCs)	BM-MSCs, AD-MSCs, UC-MSCs, Wharton’s jelly, DPSCs	● Paracrine secretion (trophic factors, EVs) Immunomodulation (M1 → M2 polarization, Treg expansion) ● Mitochondrial transfer and bioenergetic rescue ● BBB protection and edema resolution (AQP4 modulation) ● Angiogenesis, neurogenesis, oligodendrogenesis	**IV:** minimally invasive, pulmonary first-pass (<2% brain engraftment), best for systemic immunomodulation. **IA:** higher initial cerebral engraftment, risk of microemboli, requires interventional expertise. **IT:** CSF distribution, less invasive than IC. **IN:** non-invasive, bypasses BBB, low efficiency for whole cells → suited for EVs/secretome.	**Subacute (days–weeks)**: most common window; permissive microenvironment (M2 shift, SDF-1α). **Chronic phase:** plasticity promotion; feasible but less studied. Acute (<48 h) less favorable (hostile milieu, TREASURE trial negative).	● Low immunogenicity (MHC-II low) ● “Off-the-shelf” allogeneic potential ● Excellent safety profile (no tumorigenesis in trials) ● Multiple accessible sources (UC, AD, BM)	● Low cerebral engraftment after IV delivery ● Variable potency across donors/passage ● Limited direct neuronal integration ● Lack of standardized potency assays	Choudhery et al., 2025; Jiang et al., 2025; Kaur et al., 2021; Nguyen et al., 2025; Sarmah et al., 2022; Shen et al., 2024
Neural stem/progenitor cells (NSCs/NPCs)	Fetal CNS, iPSC differentiation, somatic cell reprogramming (iNSCs)	● Direct neuronal replacement and synaptic integration ● Paracrine trophic support (GDNF, BDNF) ● Promotion of endogenous neurogenesis and remyelination ● Modulation of glial scar	**IC (intracerebral)**: stereotactic injection into infarct cavity/parenchyma – maximizes local cell density, ideal for structural repair. **IT:** alternative for broader distribution via CSF. ● IV/IA rarely used for NSCs due to low engraftment and safety concerns.	**Chronic phase (months+)**: stable cavity, environment permissive for integration; subacute explored but invasive delivery preferred after lesion stabilization.	● High potential for neural circuit reconstruction ● Capacity for true neuronal replacement ● Synaptic integration into host networks	● Ethical/logistical constraints (fetal tissue) ● Higher theoretical tumorigenic risk (residual undifferentiated cells) ● Requires invasive IC delivery ● Stringent quality control needed (suicide genes, sorting)	Kawabori et al., 2024; Wang S. et al., 2024
Bone marrow mononuclear cells (BMMNCs)	Autologous bone marrow aspirate (unfractionated)	● Heterogeneous mixture: paracrine effects, immunomodulation ● Endothelial progenitor cells (EPCs) support angiogenesis ● Limited direct differentiation; mainly trophic/immune modulation	**IV:** standard route for autologous use, simple, safe. **IA:** investigated in some trials for higher brain delivery. No major pulmonary entrapment advantage but variable composition.	**Subacute phase:** most clinical data available (days to weeks). Also used in chronic settings but evidence less robust.	● Autologous → no immune rejection ● Simplified regulatory pathway ● Bedside preparation possible ● Low cost relative to cultured cells	● Unfractionated, highly variable cell composition ● Less defined active component (MSC, HSC, immune cells) ● Potency depends on donor age/health ● Difficult to standardize	Chang W. et al., 2025; Habib and Steinberg, 2025
iPSC-derived MSCs (iMSCs)	Differentiation of induced pluripotent stem cells (iPSCs) into MSC-like cells	● Paracrine and immunomodulatory effects similar to primary MSCs ● Secrete BDNF, VEGF, EVs; promote neuroprotection and mitochondrial bioenergetics ● Equivalent efficacy to BM-MSCs in preclinical stroke models	Same as MSCs: IV (most common), IA, IT, IN (for EVs). Scalable production enables consistent off-the-shelf availability.	Likely optimal in subacute phase based on MSC literature; further clinical validation ongoing.	● Unlimited expandability from well-characterized iPSC banks ● Highly standardized, clinical-grade manufacturing potential ● Combines MSC safety with iPSC scalability	● Long-term safety of iPSC-derived products under evaluation ● Tumorigenic risk if residual pluripotent cells remain ● Complex GMP differentiation protocols	Arakawa et al., 2023; Kawatani et al., 2025

MSCs, mesenchymal stem/stromal cells; NSCs/NPCs, neural stem/progenitor cells; BMMNCs, bone marrow mononuclear cells; iPSCs, induced pluripotent stem cells; iMSCs, iPSC-derived MSCs; UC-MSCs, umbilical cord MSCs; AD-MSCs, adipose-derived MSCs; DPSCs, dental pulp stem cells; EVs, extracellular vesicles; IV, intravenous; IA, intra-arterial; IC, intracerebral; IT, intrathecal; IN, intranasal; BBB, blood-brain barrier; CSF, cerebrospinal fluid.

## 5 The next frontier: cell-free therapies and bioengineering enhancements

Recognizing the inherent risks and limitations of live cell administration, research now concentrates efforts on two primary strategies: leveraging cell-free therapeutic components and engineering cells for enhanced efficacy.

### 5.1 Extracellular vesicles and exosomes: the acellular revolution

Because stem cells primarily achieve therapeutic efficacy via paracrine secretion, research focuses intently on extracellular vesicles (EVs), which are lipid-bilayer nanoparticles (e.g., exosomes and microvesicles) harboring proteins, lipids, and nucleic acids (miRNA, mRNA, and DNA). Mesenchymal stem/stromal cell-derived EVs (MSC-EVs) and neural stem/progenitor cell-derived EVs (NSC-EVs) replicate key therapeutic functions (immunomodulation, neuroprotection, angiogenesis, neurogenesis). They offer distinct benefits over live cells (Bang and Kim, 2022; Guo et al., 2021), including superior safety (eliminating risks of tumorigenicity, vascular occlusion, or lineage contamination), lower immunogenicity, and suitability for scalable manufacturing: purification, characterization, lyophilization, and stable storage as “off-the-shelf” biologics. Furthermore, their nanoscale dimension aids the traversal of biological barriers, notably the BBB. EVs are highly tunable: bioengineering permits enhanced therapeutic efficacy through exogenous cargo loading (e.g., miR-124 for neurogenesis, drugs) or membrane decoration with targeting ligands (e.g., the RVG peptide) to improve homing to the injured brain (Yang et al., 2022; Zhou et al., 2024). Compelling preclinical data show that EV administration reduces infarct volume, improves functional recovery, and activates protective pathways (e.g., PI3K/Akt/mTOR) (Dehghani et al., 2021; Wang et al., 2025). EVs also promise utility as biomarkers, given that their plasma molecular cargo reflects the cerebral pathological state and treatment response (Chang C. et al., 2025).

#### 5.1.1 Standardization and characterization challenges

Clinical translation of EV-based therapies requires adherence to evolving standardization frameworks. The MISEV2023 guidelines (Minimal Information for Studies of Extracellular Vesicles) provide a foundational consensus on nomenclature, isolation, characterization, and reporting, emphasizing quantitative metrics such as particle-to-protein ratio and the expression of canonical EV markers (CD9, CD63, CD81) (Berlet et al., 2021). However, translation to Good Manufacturing Practice (GMP) remains challenging. Current isolation methods–including ultracentrifugation, polymer-based precipitation, and microfluidics–each present trade-offs between purity, yield, and scalability. Ultracentrifugation remains the gold standard but suffers from low throughput and potential shear-induced damage; precipitation methods are scalable but co-isolate contaminating lipoproteins; microfluidics offer high purity but require further validation for large-scale production. Achieving harmonized manufacturing standards is a prerequisite for regulatory approval.

#### 5.1.2 Potency assays and functional characterization

Unlike living cells, EVs cannot be assessed by viability or proliferation. Establishing potency assays that correlate with *in vivo* efficacy is therefore a critical regulatory bottleneck. Current approaches include functional readouts such as microglial M2 polarization, endothelial tube formation, or neurite outgrowth *in vitro*, complemented by quantitative profiling of EV-associated miRNAs or proteins. The absence of validated, release-ready potency assays remains a major barrier to clinical translation, necessitating continued method development and alignment with regulatory expectations.

#### 5.1.3 Biodistribution, pharmacokinetics, and brain targeting

Systemically administered EVs are rapidly cleared by the mononuclear phagocyte system, with a circulation half-life of minutes to hours. Biodistribution studies consistently show predominant accumulation in the liver, spleen, and lungs, with limited brain uptake. However, EV-mediated effects often persist beyond their clearance, suggesting an “initiator” mechanism that triggers host repair cascades rather than requiring sustained presence. To enhance brain targeting, surface engineering strategies–such as conjugation of RVG peptide, cRGD, or other brain-homing ligands–have been shown to improve cerebral accumulation and functional outcomes in preclinical stroke models. Understanding the relationship between administration route, biodistribution, and therapeutic efficacy is essential for rational EV-based trial design.

#### 5.1.4 Clinical trial landscape

Several phase I/II trials have evaluated MSC-derived EVs in ischemic stroke, with preliminary results indicating favorable safety profiles and signals of functional improvement. Registered studies (e.g., NCT03384433, NCT05060107) are exploring EV administration in acute and subacute stroke settings. However, no phase III trials have been completed to date. The off-the-shelf, lyophilizable nature of EVs positions them as particularly attractive for acute stroke interventions, where time-sensitive delivery is critical. Moving forward, harmonized manufacturing standards, validated potency assays, and well-designed randomized controlled trials are essential to establish EV-based therapies as a viable clinical option.

### 5.2 Genetic engineering of stem cells

Genetic engineering strategies are widely explored to potentiate intrinsic stem cell therapeutic capacity. Beyond enhancing paracrine effects, genetic modification directly addresses the survival bottleneck. Overexpression of anti-apoptotic genes such as Bcl-2 or stress resistance factors like HIF-1α has been shown to improve graft survival in preclinical stroke models, thereby extending the therapeutic window of transplanted cells. Approaches include the overexpression of key trophic factors [e.g., brain-derived neurotrophic factor (BDNF), glial cell line-derived neurotrophic factor (GDNF), or vascular endothelial growth factor (VEGF)] to amplify paracrine effects. For instance, genetically modified mesenchymal stem/stromal cells (MSCs) overexpressing BDNF demonstrate superior efficacy in animal stroke models (Wang X. et al., 2024). Alternatively, enhancing cellular homing is achieved by engineering overexpression of specific chemokine receptors, such as CXCR4. Since CXCR4 binds stromal cell-derived factor-1α(SDF-1α)–which is upregulated in ischemic tissue–this mechanism improves migration from the circulation to the injury site (Achón Buil et al., 2023). Finally, gene editing tools like CRISPR/Cas9 are employed to create universally compatible “off-the-shelf” products. This endeavor entails the targeted knockout of Major Histocompatibility Complex (MHC) molecules in allogeneic induced pluripotent stem cell (iPSC)-derived cells to evade immune rejection, a promising approach currently under development (Achón Buil et al., 2024)

### 5.3 Preconditioning and priming strategies

Preconditioning and priming strategies involve applying sub-lethal stress to stem cells *in vitro*, thereby upregulating survival and reparative programs prior to transplantation. Specifically, hypoxic preconditioning (culturing cells under 1%–3% O2 to simulate ischemia) upregulates HIF-1α and associated factors, including survival factors (VEGF and erythropoietin) and homing receptors. This enhanced profile enhances cellular resilience within the hostile post-stroke milieu. Conversely, pharmacological or cytokine priming–using agents such as melatonin, low-dose lipopolysaccharide (LPS), or interferon-gamma (IFN-γ)–augments immunomodulatory capacity and trophic factor secretion profiles. These preconditioning strategies address the survival bottleneck by upregulating cytoprotective pathways such as Akt, Bcl 2, and HIF 1α, thereby increasing the proportion of cells that withstand the acute post transplantation period. However, even with optimized preconditioning, cell persistence remains limited to weeks, indicating that complementary strategies such as scaffold based retention or genetic modification are needed when sustained cell presence is desirable.

### 5.4 Biomaterial scaffolds and combination therapies

Scaffold-assisted delivery represents a bioengineering strategy to improve cell therapy outcomes. This approach is particularly promising for overcoming the survival bottleneck. Biomaterial scaffolds provide a supportive three dimensional microenvironment that protects transplanted cells from anoikis, modulates local inflammation, and can sustain trophic factor release. Preclinical studies have shown that encapsulation in hydrogels or injectable scaffolds extends graft survival from days to weeks or months, enabling a more sustained therapeutic window, especially for chronic stroke where prolonged paracrine activity is sought. Embedding cells in hydrogels or injectable scaffolds, such as those based on hyaluronic acid or synthetic polymers, provides three-dimensional structural support. This protects cells from host immune clearance, enhances retention at the delivery site, and can enable the controlled release of trophic factors. Some advanced scaffolds are designed to be conductive or include micropores to facilitate vascular ingrowth (Kaiser et al., 2022; Wu et al., 2025). Another critical avenue is the synergistic combination of stem cell therapy with established modalities. Physical rehabilitation, the cornerstone of stroke recovery which drives activity-dependent plasticity, shows strong preclinical and emerging clinical evidence for synergy when combined with stem cell therapy. Structured rehabilitation in an enriched environment with task-specific training likely provides the necessary neural activity to guide and strengthen the new connections fostered by the transplanted cells (Berlet et al., 2021, 2022). Additionally, co-administering stem cells with adjunct neuroprotective agents can help create a more favorable microenvironment for cell survival and function. Examples include the use of Tanshinone IIA delivered via nanoparticles to improve neural stem/progenitor cell (NSC) survival (Kaiser et al., 2022) or redox nanoparticles to scavenge reactive oxygen species (ROS) and protect transplanted cells (Hirata et al., 2023).

## 6 Clinical translation: regulatory, manufacturing, and reimbursement barriers

Beyond scientific challenges, the clinical adoption of stem cell therapies for stroke critically depends on success in regulatory approval, scalable manufacturing, and sustainable reimbursement.

### 6.1 Regulatory classification and manufacturing scalability

Regulatory frameworks vary significantly across jurisdictions. In the United States, the distinction between minimally manipulated 361 HCT/Ps and 351 biologics dictates the development pathway; most expanded allogeneic products fall under the 351 framework. The European Union categorizes engineered cell products as Advanced Therapy Medicinal Products (ATMPs). Extracellular vesicle (EV)-based therapies are typically classified as biologic drugs, requiring distinct Good Manufacturing Practice (GMP) standards that remain under development. The combination of cells with biomaterial scaffolds adds further complexity as combination products.

Manufacturing scalability remains a critical barrier. Autologous therapies face high per-patient costs, lengthy lead times, and batch-to-batch variability. Allogeneic products enable “off-the-shelf” availability but require substantial upfront investment in master cell banks and robust quality systems. For EV-based therapies, GMP-compliant production yields often fall short of clinical dosing requirements, and purification methods lack standardization. Across product classes, the absence of validated potency assays that correlate with *in vivo* functional outcomes remains a fundamental regulatory gap.

### 6.2 Demonstrating efficacy and achieving reimbursement

Demonstrating efficacy to regulatory standards is complicated by the inherent heterogeneity of stroke–including lesion characteristics, patient age and comorbidities, and timing of intervention–coupled with variability in cell product characteristics. The negative TREASURE trial exemplifies the challenge of applying a single protocol to a heterogeneous patient population within a narrow therapeutic window. Adaptive and biomarker-driven trial designs that incorporate pre-specified patient stratification offer a promising alternative to traditional “one-size-fits-all” pivotal trials.

Even with regulatory approval, sustainable reimbursement is essential for clinical adoption. Cell and EV therapies are inherently costly to manufacture, conflicting with conventional reimbursement models designed for chronic medications rather than single, potentially disease-modifying, high-cost interventions. Payers require evidence of cost-effectiveness–for stroke, this means demonstrating long-term reductions in disability-related costs, including institutional care and loss of productivity. Future trials must therefore incorporate health economic endpoints, patient-reported outcomes, and long-term healthcare utilization data to support value-based reimbursement models.

Building upon these translational barriers, the following discussion outlines a prioritized roadmap to address the most critical scientific and strategic gaps in the field over the next decade.

## 7 Discussion

Beyond the regulatory, manufacturing, and reimbursement barriers outlined above, additional scientific and strategic challenges continue to impede the routine clinical application of stem cell therapies for stroke. In this discussion, we articulate a set of prioritized, action-oriented positions that we believe should guide the field over the next decade.

We argue that the single highest priority for the next decade is not identifying a single “best” cell type, but rather establishing harmonized manufacturing standards and validated potency assays. Product heterogeneity, including donor variability, passage number, and inconsistent isolation protocols, remains a fundamental barrier. We contend that the field must converge on minimal potency criteria that correlate with *in vivo* functional outcomes (e.g., microglial M2 polarization capacity for mesenchymal stem/stromal cell products) and implement these as release criteria before clinical use. Concurrently, scalable Good Manufacturing Practice (GMP) production, particularly for extracellular vesicle (EV), based products, requires technological consolidation around closed-system bioreactors and purification methods that balance purity, yield, and regulatory compliance.

A central strategic question is whether cell-free therapies will replace live cell therapies. Our view is that these platforms are complementary, with their optimal use defined by timing, mechanism, and therapeutic goal. Live cells, particularly neural stem/progenitor cells, retain unique advantages when structural integration or sustained paracrine activity is required, scenarios most relevant to chronic stroke. Conversely, EVs offer superior safety, lower immunogenicity, and true “off-the-shelf” accessibility, making them better suited for acute interventions. We propose positioning these modalities along the post-stroke timeline: EV-based therapies prioritized in the acute window, and live cell therapies reserved for subacute and chronic phases where sustained tissue remodeling is pursued.

The variable efficacy observed in clinical trials, epitomized by the negative TREASURE trial, leads us to conclude that the next generation of pivotal trials must be biomarker-driven. We propose a three-phase biomarker approach. First, pre-selection: baseline neuroimaging and plasma inflammatory profiles to exclude patients with low likelihood of response. Second, stratification: randomization stratified by immune phenotype or genetic variants (e.g., BDNF Val66Met) to reduce outcome variance. Third, surrogate endpoints: Phase II studies incorporating advanced MRI metrics and (Helsper et al., 2023; Liu et al., 2025) EV-based molecular biomarkers as early efficacy surrogates. We contend that without such a biomarker-integrated framework, even biologically active therapies risk failure in heterogeneous, underpowered studies.

Beyond scientific and manufacturing priorities, clinical adoption requires regulatory clarity and sustainable reimbursement. Regulatory classification remains uncertain, particularly for EV products, which typically fall under biologic drug frameworks. We emphasize that early regulatory engagement is essential, and that future trials must be co-designed with health economists to incorporate long-term healthcare utilization and quality-of-life metrics. Demonstrating cost-effectiveness through reduced long-term care dependency will be critical for reimbursement.

In summary, we envision that the next decade will be defined not by a single winning technology, but by strategic alignment of product choice, biomarker-guided trial design, manufacturing standardization, and regulatory-reimbursement integration. It is through such concerted effort that stem cell-based and cell-free therapies can transition from scientific promise to an established component of comprehensive stroke rehabilitation.

## 8 Conclusion

Stem cell therapy for stroke has evolved beyond simple cell replacement into a sophisticated paradigm that orchestrates intrinsic neural repair through immunomodulation, trophic support, and enhanced plasticity. Despite consistent safety profiles in clinical trials, efficacy remains highly variable, a divergence rooted in protocol heterogeneity and patient-specific factors. The field now emphasizes two complementary approaches: bioengineering enhanced cellular products and the development of acellular, extracellular vesicle-based therapies. Realizing the full therapeutic potential requires rigorous deployment of large, standardized, biomarker-stratified trials coupled with interdisciplinary collaboration to define optimal patient cohorts, delivery protocols, and product specifications. Only through such concerted investigation can stem cell-based approaches achieve status as a validated component of comprehensive stroke rehabilitation, thereby restoring functional recovery for survivors.
